# Impact of implementing a category 1 external quality assurance scheme for monitoring harmonization of clinical laboratories in Spain

**DOI:** 10.1515/almed-2020-0008

**Published:** 2020-03-19

**Authors:** Carmen Ricós, Pilar Fernández-Calle, Fernando Marqués, Joana Minchinela, Ángel Salas, Cecília Martínez-Bru, Beatriz Boned, Rubén Gómez-Rioja, Marià Cortés, Elisabet González-Lao, JV García-Lario, Xavier Tejedor-Ganduxé, Sandra Bullich, Montse Ventura, Margarida Simón, Carlos Vilaplana, Ricardo González-Tarancón, Mª Pilar Fernández-Fernández, Francisco Ramón-Bauzá, Zoraida Corte, Mª Antonia Llopis, Jorge Díaz-Garzón, Carmen Perich

**Affiliations:** Comité de Programas Externos de la Calidad, SEQC^ML^ , Barcelona, Spain; Comisión de Calidad Analítica, SEQC^ML^ , Barcelona, Spain; Plaza Gala Placidia 2, ático, Barcelona, Spain

**Keywords:** analytical performance specifications, biological variation, external quality assurance schemes, harmonization, state of the art

## Abstract

**Background:**

The objective of the present study was to examine the evolution of the analytical performance specifications (APS) used in External Quality Assurance (EQA) schemes, as well as the efficacy of a category 1 EQA scheme in monitoring the harmonization of clinical laboratory results in Spain.

**Methods:**

A review of the literature on the types of quality specifications used in schemes in other countries and their evolution was performed. In addition, a comparative analysis of the potential impact that different APS from eight countries had on clinical decision-making was made based on three measurands: sodium, thyroid-stimulating hormone (TSH), and activated partial thromboplastin time (aPTT).

**Results:**

Harmonization of analytical methods was demonstrated by assessing whether average results deviated from the certified reference value of control materials within the APS derived from biological variation (BV). The APS used in EQA have evolved from state-of-the-art models to BV. Poor clinical decision-making would occur if the results accepted by some APS were applied.

**Conclusions:**

In Spain, only 2 of the 18 measurands studied are considered to be well harmonized. Closer collaboration between laboratories and analytical system providers would be required to resolve discrepancies.

## Introduction

The main objective of clinical laboratories is to provide useful information for adequate clinical decision-making. In this line, standardization and harmonization of analytical procedures are among the current priorities of laboratory medicine. On the one hand, harmonization aims to guarantee the comparability of test results between different laboratories and healthcare systems. In this context, standardization is a further step that aims to make test results traceable to International System (IS) units by using a reference method or a standard calibration material.

The test results obtained in different laboratories should be interchangeable, i. e., they should be *standardized* (in the case of measurands with available calibration standards and reference methods) or *harmonized* (in the absence of such standards). This would contribute to adequate clinical decision-making, regardless of the laboratory that performs the analytical tests.

Harmonization of results in laboratory medicine requires participating in an External Quality Assurance (EQA) scheme and collaborating with relevant organizations, including *in vitro* diagnostic product manufacturers, the reference laboratories acknowledged by the Joint Committee for Traceability in Laboratory Medicine (JCTLM) [1], and clinical laboratories [2], [3], [4].

In 1994, The International Federation of Clinical Chemistry and Laboratory Medicine (IFCC) Committee on Analytical Quality specified that comparisons between laboratories should be designed and carried out to ensure one or more of the following aspects [5]: evaluation of the services provided by the participant and the analytical methods used; evaluation of *in vitro* diagnostic devices; and continuous education, training, and support.

A crucial element of an EQA scheme is the rationale for the analytical performance specifications (APS) and the type of APS used [6], [7]. There is a relationship between the quality of APS expected to be achieved (ideal, optimum, desirable, and minimum) and the type of APS used (e. g., optimum limit derived from biological variation [BV], 20th percentile of participants, or deviation index <1), irrespective of the clinical use indicated [8].

The capacity to evaluate laboratory performance depends on the design of the EQA scheme. Five categories have been described, ranging from 1 to 5 in decreasing order of capacity [9]. Category 1 schemes use commutable control materials with human specimens, with target values assigned by reference methods or materials, in which replicates of control materials are tested. These schemes possess the highest evaluation capacity.

Following these guidelines, in 1994, the Spanish Society of Laboratory Medicine (SEQC^ML^) addressed the development of EQA schemes. The criteria used by SEQC^ML^ were based on the clinical use of laboratory data (derived from BV) in the evaluation of methods carried out at the end of each cycle. In 2001, the evaluation of individual results for each participant based on BV was included as well. Throughout the early years, all the EQA schemes of the SEQC^ML^ were category-4 schemes (i. e., non-commutable controls composed of human or bovine specimens, with values assigned by consensus agreement between the methods used and replicate analyses of control samples). The totality of EQA schemes were aimed at monitoring laboratory performance. Since 2015, the SEQC^ML^ also offers a category 1 scheme aimed at promoting harmonization, which involves obtaining results that are interchangeable and traceable to reference standards [6], [10].

The objective of this study was to examine the evolution of the APS used in EQA schemes of different countries, as well as the efficacy of a category 1 scheme in monitoring the harmonization of clinical laboratory results in Spain.

## Materials and methods

This study was performed using the results obtained in the category 1 EQA scheme distributed in Spain during a 4-year period (2015–2018).

The methodology used involved:

(1) Reviewing the scientific literature to examine the evolution of the types of APS used in EQA schemes in different countries.
(2) Comparing the potential impact of applying the different APS to EQA schemes on clinical decision-making. For this purpose, the APS used in the category 1 scheme of the SEQC^ML^, as well as those used in eight schemes in other countries, were applied to the theoretical test results for sodium, thyroid-stimulating hormone (TSH), and activated partial thromboplastin time (aPTT).
(3) Describing the degree of harmonization of laboratory test results in Spain by evaluating the results of the category 1 scheme of the SEQC^ML^ during four consecutive cycles (from 2015 to 2018). To such purpose, the percentage deviation of the mean value obtained for each peer group with respect to the certified reference value is calculated for each control material. Peer groups are constituted by a combination of the analytical method, the traceability of the calibrator and the instrument used. For instance, for the determination of sodium content, a peer group would be formed by every laboratory using the indirect ion-selective electrode (ISE) method traceable to the reference material NIST-SRM 956 in an Abbott Architect automatic analyzer.

Results are considered to be standardized if percentage deviations from the certified reference material are within the APS derived from BV for systematic error (optimum, desirable or minimum, depending on the measurand).

## Results

**Figure 1:**
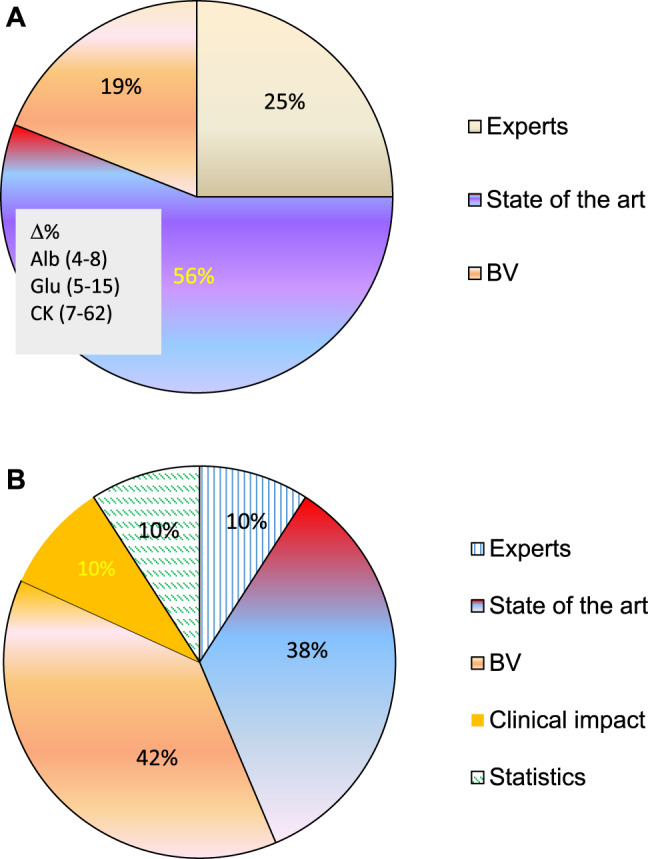
Evolution of the type of APS used in EQA schemes. (A) 16 Europeans EQA schemes, 1996. (B) 10 international EQA schemes, 2017.

Figure 1A, B shows the evolution in the type of APS used in EQA schemes according to the literature. In 1996, a first survey conducted among 16 European EQA schemes revealed that most organizations used the state of the art as APS (existing performance at that time) (Figure 1A). Based on this criterion, notable discrepancies were observed, namely: some EQA accepted 4–8% deviations from the target value for serum albumin; whereas 5–15% deviations were accepted for glucose, and accepted deviations for creatine kinase (CK) ranged from 7 to 62% for [11]. In 2017, 21 years later, a survey submitted to EQA scheme organizers in different countries revealed that most of them used specifications based on BV [7], as suggested in the first Strategic Conference of the European Federation of Clinical Chemistry and Laboratory Medicine (EFLM), held in Milan in 2014 [12] (Figure 1B).

Accordingly, BV data can be retrieved from the websites of the SEQC^ML^ [13], Westgard [14] and the EFLM [15].

Table 1 shows examples of the impact on clinical decision-making based on the type of APS used. This table displays the APS used in EQA schemes of different countries for certain measurands. For instance, a patient with a sodium value of 133 mmol/L (i. e., hyponatremia) could not receive a diagnosis in 6 of the 8 countries; a euthyroid woman could be diagnosed with hypothyroidism and unnecessarily treated in 3 of the 8 countries; and a coagulation disorder could be not detected in 3 of the 7 countries examined (one country did not send coagulation test results). However, it should be noted that this comparison is theoretical and does not take into account the actual performance of participant laboratories.

**Table 1:** Interval of potential results for a theoretical result due to the use of different APS in national EQA schemes.

Measurand	EPA Result	CLIA´19 (USA)	RILIBÄK (Germany)	UK-NEQAS (United Kingdom)	SKML (Netherlands)	NOKLUS (Norwey)	RCPAQAP (Australia)	ASQUALAB (France)	SEQC^ML^ (Spain)
Sodium	EPA	4 mmol/L	3%	2%	0.73%	2%	3 mmol/L (2%)	2.5%	1.1%
	133 mmol/L	129–**137**	130–**137**	130–**136**	132–134	130–**136**	130–**136**	130–**136**	132–134
TSH	EPA	20%	13.5%	12.5%	23.7%	12%	0.6 mU/L (15%)	20%	11.9%
	4.1 UI/L	3.28–**4.92**	3.5–4.7	3.6–4.6	3.1–**5.1**	3.6–4.6	3.5–4.7	3.28–**4.92**	3.6–4.6
aPTT	EPA	15%	10.5%	15%	4.5%	5%	–	20%	6.7%
	1.4	**1.19**–1.61	1.25–1.55	**1.19**–1.61	1.34–1.46	1.34–1.46	–	**1.18**–1.65	1.31–1.49

Values in bold indicate the accepted values that could lead to a poor clinical indication.

**Figure 2:**
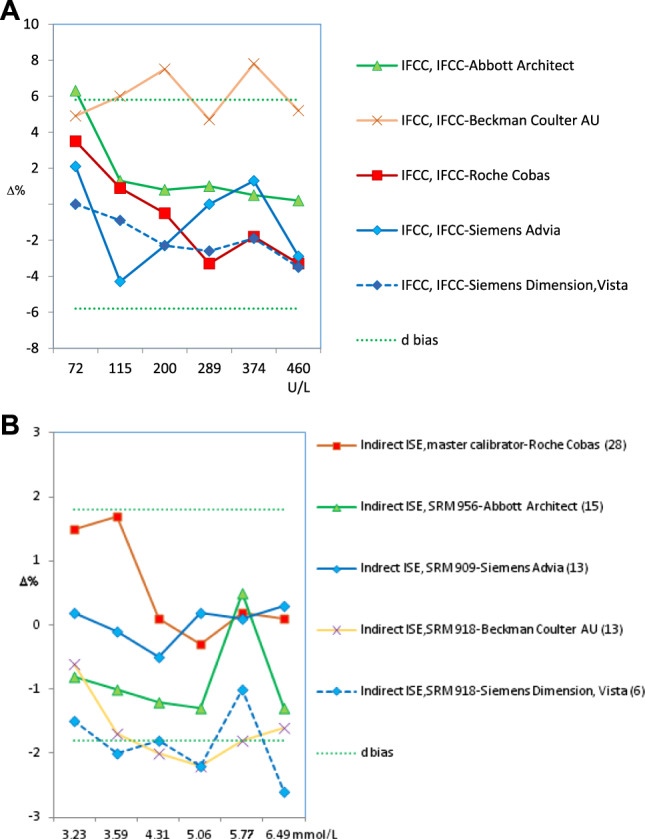
Bias obtained for standardized analytes. (A) CK (2018). (B) Potasium (2015–2018). Green dotted lines indicate the acceptable upper and lower bias based on BV. The bias shown may be the desirable (d) or the optimum (o). The number of participating laboratories in each comparison group is indicated in brackets.

**Figure 3:**
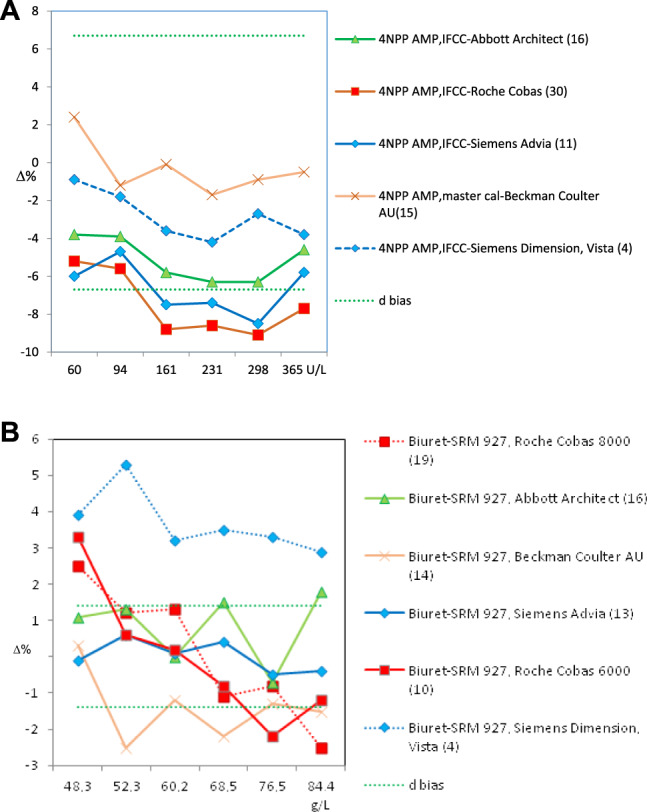
Bias obtained for non-standardized analytes according to the instrument used. (A) Alkaline phosphatase (2018). (B) Proteins (2018). Green dotted lines indicate the acceptable upper and lower bias based on BV. The bias shown may be the desirable (d) or the optimum (o). The number of participating laboratories in each comparison group is indicated in brackets.

With regard to the test results over the four cycles of the SEQC^ML^ category 1 EQA scheme (from 2015 to 2018), four types of results were obtained:

– Adequate standardization was achieved only for CK and potassium (2 of the 18 measurands included in the scheme) (Figure 2A, B).In the case of CK, all groups used the same method (recommended by the IFCC and traceable to the IFCC reference method); and all instruments produced standardized results (Figure 2A), except for Beckman Coulter AU. For determination of potassium content, all groups used the indirect ISE method, with different types of traceability. The percentage deviation from the overall mean (for six controls) obtained over a 4-year period remained within the APS interval in three of the six participating groups. In the remaining groups, a clear improvement could be observed. Therefore, almost all groups reached the APS in the last year (Figure 2B).
– Standardization for alkaline phosphatase and protein was not achieved due to the instrument used (Figure 3A, B, respectively).In both cases, all groups used the same methodology, i. e., the method recommended by the IFCC, traceable to the IFCC reference method for alkaline phosphatase, and the Biuret method traceable to NIST-SRM 927 for the protein.

Regarding alkaline phosphatase, two of the five participating groups (i. e., Roche Cobas and Siemens Advia) reported results below the lower limit of the APS (6.7%), with deviations of −8.3% and −7.8%, respectively. Unfortunately, they were the largest groups in our scheme. Braga et al. [16] observed the same negative deviation in users of Roche Cobas in a category 1 scheme performed in Italy. In the case of the protein, only Siemens Advia users achieved good results in 2018 for all control samples, whereas Siemens Dimension/Vista users obtained high results. The users of the remaining instruments obtained irregular results in the 4-year period.

**Figure 4:**
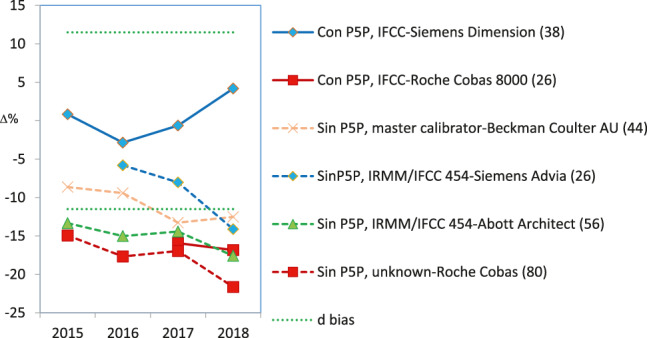
ALT. Bias depends on the analytical method used. Green dotted lines indicate the acceptable upper and lower bias based on BV. The bias shown may be the desirable (d) or the optimum (o). The number of participating laboratories in each comparison group is indicated in brackets.

**Figure 5:**
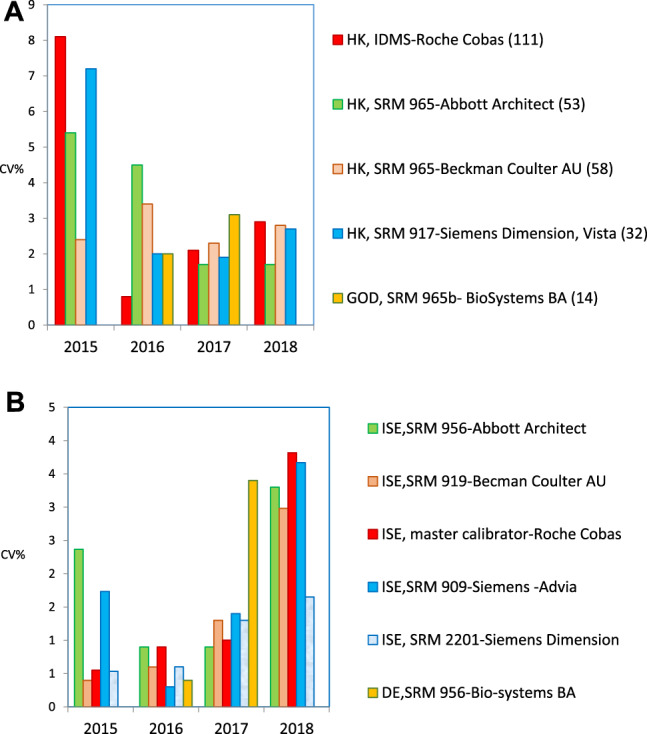
Evolution of inter-laboratory imprecision (CV%) for glucose and sodium. (A) Glucose. (B) Sodium.

– Due to the method used, standardization was not achieved for *α*-amylase, creatinine, *γ*-glutamyltransferase (GGT), lactate dehydrogenase (LDH), alanine aminotransferase (ALT) and aspartate aminotransferase (AST). In the measurement procedure of *α*-amylase, the maltotriose substrate method yielded low results. As an example, Figure 4 illustrates the case of ALT, in which the method that does not add pyridoxal phosphate (PLP) showed a negative deviation (−18%), regardless of the instrument or traceability applied. The same deviation was observed by Goossens et al. [17] in a category 1 scheme performed in Belgium. Notably, for ALT/AST, all providers in our country offer a false method “traceable to the IFCC reference method” without the obligation of adding P5P, a decision that is left to the user's discretion. This incorrect practice affected 48% of the participants in the scheme and had a very negative impact on standardization. Conversely, high results were consistently achieved for creatinine using the Jaffe method, for GGT using the substrate <4 mmol/L method, and for LDH using the reverse pyruvate–lactate (P–L) method.
– Standardization was not achieved in seven cases that need to be further discussed with providers because they produced irregular bias and imprecision results: bilirubin, calcium, chloride, glucose, magnesium, sodium, and urate. For instance, determination of glucose showed improved inter-laboratory coefficients of variation (CV) (annual average for six controls) that started around 8% for some methods and decreased to 2–3% for all methods, which is a normal and expected evolution (Figure 5A). Conversely, sodium shows an illogical inter-laboratory CV increase over the years (1.0–3.5%) (Figure 5B). Regarding the bias, none or very few participating groups showed results within the APS in the 4-year period.

## Discussion

Participating in category 1 EQA schemes has a two-fold advantage. First, commutable control materials have the same behavior with analytical methods as human samples. Hence, the indicators of imprecision and bias obtained (as well as the inaccuracy of individual results, not discussed in this study) can be extrapolated to patients' results.

Moreover, given that values have been assigned using certified reference methods, deviation is calculated in relation to the real value and, thus, the real bias can be estimated.

In addition, these types of schemes enable comparison of results between organizations from different EQA schemes, as all the results were evaluated using the same specifications, i. e., derived from BV. Accordingly, these specifications are related to the clinical use of laboratory tests, have fixed limits independent from the analytical system used and, consequently, can be shared with other laboratories [18]. When non-uniform APS criteria are used, wrong clinical decisions can be made, as shown in Table 1.

Due to the fact that our results are consistent with other EQA schemes, potential causes for the lack of harmonization can be proposed. For instance, using different analytical procedures for the same analyte, as described in this study for creatinine, amylase with maltotriose as substrate, ALT/AST without P5P, or LDH using the reverse P–L method. Other causes have been suggested elsewhere, including a possible batch-to-batch variation of calibrators, inappropriate models for the calibration curve, and incorrect assignment of values to the calibrators [19].

The use of APS derived from BV has evolved favorably over the years. The use of these specifications has advantages and drawbacks. The main limitation is that no BV data are yet available for all the measurands analyzed in clinical laboratories. The main advantage is that BV concerns the clinical use of laboratory tests for patient care. BV involves fixed limits independent of the analytical systems that can be shared with other laboratories, clinicians and users of the healthcare system.

The results presented in this article and in another article also published in this issue [20] lead to the following recommendations:

– Clinical laboratories should participate in a category 1 EQA scheme in order to verify that (1) the results obtained in a single analysis of patients' samples are accurate; (2) the analytical method used is standardized and traceable to reference standards; and (3) the potential bias of their measurements is acceptable from the perspective of BV, and thus share reference intervals with other laboratories that satisfy the same condition, or with the scientific literature.
– Additionally, clinical laboratories can regularly participate in EQA schemes from any other category in order to (1) monitor that their performance is maintained over time and can be compared with other laboratories using the same method or instrument; (2) evaluate measurands for which no reference materials or methods exist; and (3) determine which analytical methods and analyzers yield better results.
– The above-mentioned points do not exclude the necessity of monitoring internal analytical quality daily, which guides the acceptance or refusal of analytical series and helps obtain the corresponding imprecision indicator.
– When the state of the art is used as APS, the laboratory must try to compare itself with the best possible performance [21], e. g., 20th percentile instead of average performance (i. e., 50th percentile) or the most frequent (90th percentile). As long as optimal performance is not achieved, the laboratory should focus on this purpose.
– Another interesting aspect of EQA schemes lies in their utility to improve the harmonization of analytical methods among manufacturers and monitor the efforts made for such purpose. This article, along with scheme evaluations and meetings with the manufacturers, illustrates such utility for the healthcare system, apart from the direct benefit for the laboratory.

In summary, the category 1 EQA scheme distributed in Spain during a 4-year period demonstrates that the results in our country are not yet adequately standardized for all the studied measurands. Closer collaboration between laboratories and analytical system providers would be required to resolve inconsistencies.

EQA scheme providers should use the specifications recommended in the consensus conference of Milan in order to make sure that the results of the participating laboratories lead to the correct diagnosis and follow-up of patients. Moreover, comparing their results with other EQA would be convenient to correctly define the state of the art and continue developing category 1 schemes.
